# Physical examination tests in the acute phase of shoulder injuries with negative radiographs: a diagnostic accuracy study

**DOI:** 10.1186/s12891-025-08754-1

**Published:** 2025-06-03

**Authors:** Martine Enger, Malte Schmidt, Lars Nordsletten, Stefan Moosmayer, Are Hugo Pripp, Knut Melhuus, Jens Ivar Brox

**Affiliations:** 1 Division of Orthopaedic Surgery, Oslo University Hospital, Postboks 4959 Nydalen, Oslo, 0424 Norway; 2 Institute of Clinical Medicine, University of Oslo, Oslo, Norway; 3 Department of Orthopaedic Surgery, Martina Hansens Hospital, Sandvika, Norway; 4 Oslo Centre of Biostatistics and Epidemiology, Oslo University Hospital, Oslo, Norway; 5 Department of Physical Medicine and Rehabilitation, Oslo University Hospital, Oslo, Norway

**Keywords:** Shoulder, Diagnostic accuracy, Physical examination, Rotator cuff, Acute shoulder injury

## Abstract

**Background:**

Rotator cuff tears may easily be missed in patients with acute shoulder trauma. The evidence in support of shoulder physical examination tests has been considered insufficient in reviews and meta-analyses. The purpose of this study was to explore whether physical examination tests can effectively predict or rule out acute full-thickness rotator cuff tears in soft tissue shoulder injuries in emergency departments and primary health care.

**Methods:**

In a combined primary care walk-in clinic and secondary care orthopaedic emergency department, 120 consecutive patients aged ≥ 40 years with acute shoulder injury without fracture on plain x-rays were enrolled prospectively at the first follow-up within three weeks of the injury. Thirteen physical examination tests and ultrasound screening as reference standard, were performed blinded to each other.

**Results:**

The median age was 55 years, 51% were female. The prevalence of the target condition rotator cuff full-thickness tear and/or occult fracture of the insertion was 38% (*n* = 46; 38 tears and 8 occult avulsion fractures). Almost all tears involved the supraspinatus tendon (*n* = 36). The highest test accuracy was observed for the inability to abduct above 90°, resisted abduction pain and external rotation strength. The sensitivity, specificity and diagnostic odds ratio of the inability to abduct the arm above 90 ° was 84% (95% CI 69–93), 71% (95% CI 59–82) and 12.9 (95% CI 4.8–34.2), respectively, and 66% (51–80), 86% (77–93) and 12.4 (5.0–30.8) for external rotation strength assessed by the small finger test. Combining the inability to abduct above 90° and weakness in external rotation improved the sensitivity to above 90% and the diagnostic odds ratio to above 22, but specificity decreased.

**Conclusions:**

The present study suggests that two simple tests, the inability to abduct above 90° and weakness in external rotation may effectively predict full-thickness tears of the supra- and infraspinatus and/or occult fracture at their insertion in the acute phase of soft tissue shoulder injuries. The test combination may be useful for selecting patients for advanced imaging and for diagnostic purposes when such imaging is not available.

**Trial registration:**

The Norwegian Regional Ethics Committee South East (2015/195) on 24th March 2015, and retrospectively registered on ClinicalTrials.gov (NCT02644564) on 31st December 2015.

**Supplementary Information:**

The online version contains supplementary material available at 10.1186/s12891-025-08754-1.

## Introduction

Shoulder pain is reported to be the third most common musculoskeletal complaint in the adult population [1]. It is difficult to estimate how common shoulder pain is as the 1-year prevalence has been reported to range from 4.7 to 46.7% [2], whereas a more recent Norwegian survey reported this at 55% [3]. Bearing the diagnostic uncertainties in mind, rotator cuff tendinopathy has been considered to be the most common cause of shoulder pain based on clinical examination [4–7], whereas 34% of primary care patients reported a traumatic incident precipitating the complaint [4].

Patients with acute shoulder traumas are normally screened with plain radiographs in emergency departments and general practice. If the images show no sign of skeletal injury, patients are in many cases discharged without further follow-up or imaging [8–12]. Thus, previous studies have shown that rotator cuff tears may easily be missed [8–10]. The evidence in support of recommending physical examination tests for rotator cuff disease has been weak or inconclusive in systematic reviews and meta-analyses [13–18]. Also, diagnostic validity studies recruiting non-referred patients examined by non-specialists have been called for [15, 16].

In the absence of reliable shoulder tests, screening of all acute shoulder injuries for rotator cuff tears by MRI or ultrasound is not a viable option. First, it is expensive and time consuming. Second, the radiological findings may simply unveil pre-existing degenerative changes unrelated to the incident. Third, there is generally poor correlation between imaging and clinical findings [19–21]. The existence of asymptomatic rotator cuff tears or abnormalities is well established, with prevalence estimates increasing with advancing age up to 65% in the general population over 80 years [19, 21–23]. A symptomatic rotator cuff tear may therefore be considered a clinical diagnosis based on patient history and physical investigation, verified by imaging [24].

In a recent study, approximately half of the shoulder injuries admitted in an emergency department were soft tissue injuries [25]. We have previously explored the interrater reliability of physical examination tests in the acute phase of shoulder injuries [26]. The aim of the present study was to assess their accuracy in predicting or ruling out acute rotator cuff tears in the first line health care.

## Materials and methods

### Study design and setting

In a prospective phase III diagnostic trial [27], 120 eligible patients were consecutively recruited at Department of Orthopaedic Emergency, Oslo University Hospital. The department is a combined primary health care walk-in clinic and secondary health care orthopaedic emergency department, treating 65,800 cases of acute injury in 2016. Patients are admitted directly without referral. The department is responsible for follow-up in patients not requiring hospitalisation. The physicians are mainly non-specialists, typically working in the department after internship.

### Participants

Patients were offered follow-up after the primary consultation according to the department`s guideline (Appendix 1). Appointments were assigned for two dedicated days every week for eligibility assessment. All patients gave written informed consent, and were enrolled between October 5th 2015 and October 17th 2016.

**Table 1:** Inclusion and exclusion criteria

Criteria	Description
Inclusion	Age ≥ 40 years
	Oslo resident
	Acute injury with concomitant onset of symptoms
	International Classification of Diseases (ICD-10) S4-diagnosis (Injuries to the shoulder and upper arm) except middle and distal third of humerus and related soft tissues
	Negative plain x-rays for signs of acute injury, or successfully reduced glenohumeral dislocation without fracture (Hill-Sachs lesions and Bankart fractures affecting < 20% of the glenoid included)
	Follow-up ≤ 21 days
Exclusion	Injury of both shoulders
	Other injury affecting shoulder symptoms or function
	Previous surgery in one of the shoulders during last 6 months
	Known rotator cuff tear on imaging
	Neck-/shoulder problems or generalized joint-/muscle pain during the last 3 months before the injury
	Other serious disease that makes participation or follow-up difficult
	Incapable of giving and or receiving adequate information, or cannot undergo normal clinical investigation.
	Does not want to participate

### Descriptive data

Age, gender, injured side, smoking status as well as time and mechanism of the injury were registered at inclusion. The patients completed the Oxford Shoulder Score (OSS) (0 (worst) to 48 (best) points) both for the week preceding the trauma and for the week preceding inclusion (after trauma) [28, 29].

### Target condition

The target condition was acute full-thickness tears of the rotator cuff, defined as a tear in a patient with an acute traumatic event with concomitant onset of symptoms in a shoulder without ongoing pain or disability at the time of the traumatic event [8, 30, 31]. We included radiographically occult fractures at the insertion site in the analysis, as a physical examination test cannot be expected to discriminate between a tear of the tendon and an avulsion of its insertion. To include fractures of the tendon insertion as a type A full-thickness rotator cuff lesion, is in accordance with the EFORT Open review classification of rotator cuff tears [32]. Occult is in the present article used about fractures which were not evident on the primary plain radiographs, but were later visualised by advanced imaging [33]. The primary radiographs were examined both by the physician in charge of the patient and a skeletal radiologist.

### Examining physicians and index tests

Four physicians had 30 min instruction and received written information on how to execute the tests. They were all licensed physicians and had from 1 to 6.5 years of experience in the department. None were specializing in or had shoulder injuries as their field of interest. They participated at random according to their work rotation. Apart from being informed about glenohumeral dislocations, they only had clinical data available by the inclusion criteria (Table 1). The physicians and patients were blinded to the results of the reference standard (ultrasound examination).

Thirteen physical examination tests and clinical signs were performed both for the injured and uninjured shoulder (Table 2). Three aspects were considered in the choice of tests: (i) Reviews and meta-analyses [13, 14, 16], (ii) the likelihood of injured patients being able to execute the test in the acute phase and (iii) the time and ease with which the tests could be performed by patients and learnt by physicians in emergency departments and general practice. As dynamometers and goniometers are not normally used in these settings, muscle strength and active range of motion were assessed pragmatically by clinical examination.

**Table 2:**
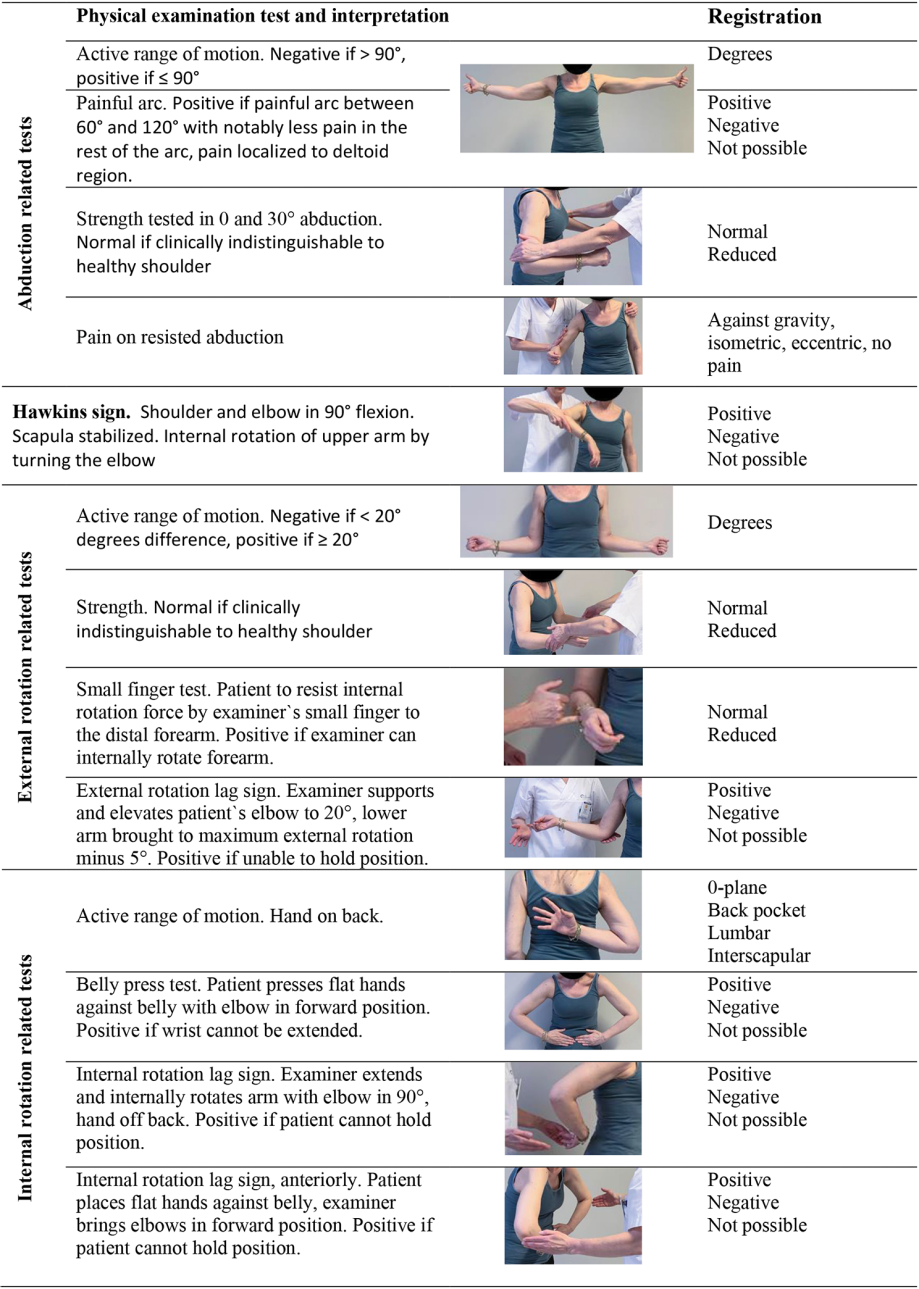
Overview of the index signs and tests. Detailed description in appendix 2

We registered the range of active abduction, and used ≤ 90 ° as test positive cut-off in the analysis [8, 34, 35]. In order not to provoke additional pain, the patients were instructed to position the upper arm with thumbs up (external rotation) during abduction. Twelve patients were stopped at 90° abduction due to the department`s recommendations for patients with recent glenohumeral dislocation and were not included in the analysis of the ability to raise the arm above 90°. The painful arc test is described both as an active and passive test [16, 36]. The performance of the test is not specified in Kessel`s original article [37]. We used the active test, instructing the patient to abduct the arm. Hawkins test was included in order to evaluate the test cluster proposed by Murrell (supraspinatus weakness, weakness in external rotation and impingement) in the present setting [38]. Abduction strength and pain in 30–40° abduction were chosen as a modified version of the supraspinatus/empty can/Jobe test [13, 14, 16], that could be performed in patients unable to elevate the arm to shoulder level.

The small finger test 1 is described Table 2. The test has been in use for more than 30 years as a more feasible alternative to the external rotation lag sign in acutely injured patients. Four new variables were generated by combining the two best abduction and external rotation tests in pairs of two. If at least one was positive, the test was registered as positive.

Inter-rater reliability was also investigated and has been published in a separate paper [26].

### Reference standard

The reference standard was ultrasound examination blinded for the results of the physical examination and vica versa. Ultrasound and MRI have similar and high diagnostic accuracy for full-thickness rotator cuff tears [39, 40]. The examination was carried out with a General Electric Logiq-e ultrasound scanner with a 12 MHz linear probe (12 L). The first author performed the scans and had completed formal courses and performed 4–6 shoulder scans per week for 1.5 years prior to the study [41].

The biceps and rotator cuff tendons were traced transversely and longitudinally with the patient`s arm in the neutral position for the biceps, subscapularis and infraspinatus tendons, the Crass position for the supraspinatus and subdeltoid subacromial bursa, and the neutral position with the shoulder extended for the rotator interval evaluation [42–44]. If a supraspinatus tear extended further than 15 mm posteriorly from the intraarticular portion of the biceps tendon, the tear was classified as involving also the infraspinatus [43]. All patients underwent bilateral ultrasound examination and bilateral physical examination on the day of inclusion, with no clinical intervention between the index tests and ultrasound examination.

Patients had follow-up and treatment according to the department`s routines. Patients considered for surgery and patients with no full-thickness tear on ultrasound but slow progress consequently had MRI in addition to ultrasound. Hence, 53 (44%) of the cohort had MRI later. There was disagreement about the target condition full-thickness tear in two cases: one was a suboptimal scan due to patient characteristics. The ultrasound scanning showed bilateral thinning of the tendon interpreted as no tear, whereas the MRI was interpreted as a full-thickness tear. In the other case ultrasound of the upper rim of the subscapularis tendon was interpreted as indeterminate, whereas MRI was described as negative. After a consensus meeting with an experienced musculoskeletal radiologist, the diagnosis was registered according to the MRI finding in these two patients.

### Statistical analysis

An a priori sample size calculation was performed based on a one-way binomial test. We assumed that the sensitivity of ultrasound for the target condition was 90%. With 80% statistical power and significance level of 5%, 120 patients were planned to be included.

As the age distribution was skewed, we have reported medians and used the Mann-Whitney U test to compare age in patients with and without a rotator cuff tear. We used the Chi-Squared test to compare the gender distribution in the same groups.

To evaluate test effectiveness, we have calculated sensitivity, specificity, positive and negative predictive values, diagnostic odds ratio (DOR), accuracy and test yield using Medcalc [45]. The test yield expresses the proportion of patients in whom a test result was achieved; a low yield indicating that many patients were incapable of performing the test or had indeterminate results. Missing data on index tests were not included in the yield calculations. The DOR reflects the entire two by two table, and is therefore commonly used to rank individual tests [14, 46, 47]. DOR and ROC curve analyses were used to assess the cut-off point for degrees of loss of abduction and external rotation. Test effectiveness results are not presented for tests of the subscapularis tendon, as only two patients had an isolated subscapularis full-thickness tear; both were small partial width tears of the upper rim.

## Results

### Participants

#### Baseline demographic and clinical characteristics

**Fig. 1:**
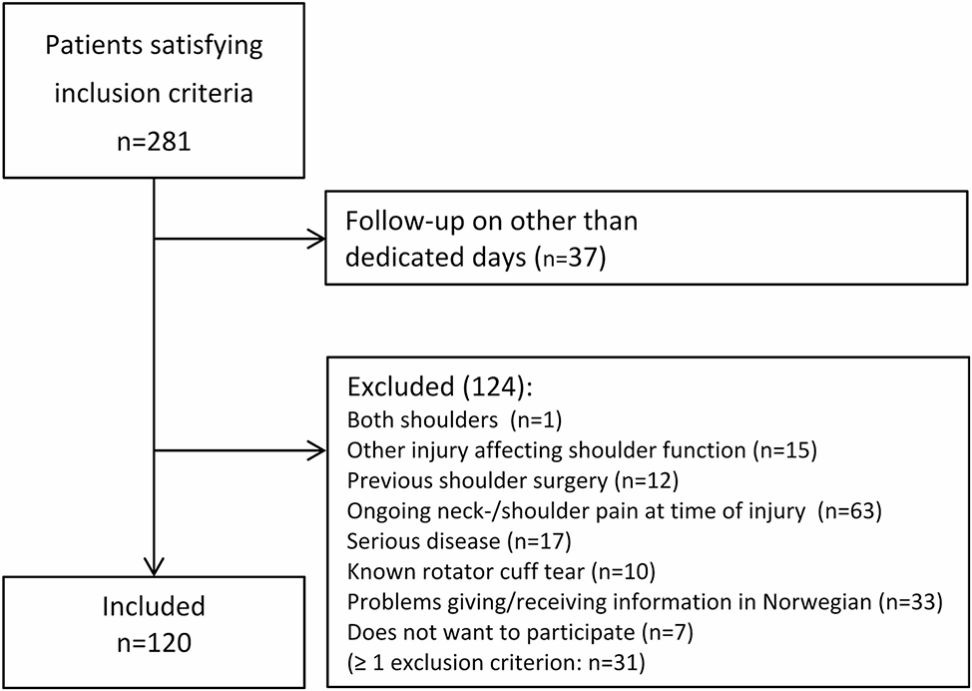
Flow of participants with inclusion and exclusion criteria

During the study period 281 patients with an acute shoulder injury were eligible and 120 included (Fig. 1).

Patient demographics and patient reported outcomes pre- and post-injury are presented in Table 3. The mean time from accident to inclusion was 12 days (Table 3). The age profile of the study population was almost identical to that of the general population (Appendix 3). Most had been injured by falls (*n* = 103, 86%) and 33% (*n* = 39) reported the injury to be sports-related. 32% were injured on roads, streets or pavements (*n* = 38). Seventeen of the participants (14%) had sustained a glenohumeral dislocation. There were no adverse events during the physical testing or ultrasound screening.

**Table 3:** Demographic data, injury mechanism and patient reported outcomes (PROMs) of study patients (*N* = 120)

Demographics and PROMs	Value
Age, years (median)	55 (interquartile range 46–66)
Sex	61 (51%) females, 59 (49%) males
Days from accident to inclusion (mean)	12 (SD 3.9)
Injured shoulder	66 (55%) right, 54 (45%) left
Smoking	101 (84%) non-smokers, 15 (13%) smokers, 4 (3%) unknown
Injury mechanism	Fall from own height 80 (67%), fall from height 25 (21%), collision 7 (6%) and acute overload 8 (7%)
OSS	25.4 (SD, 9.1)
OSS before injury *	47.7 (SD, 1.1)

*OSS* Oxford Shoulder Score: 0 worst possible, 48 best possible; *SD* standard deviation. * Filled in at inclusion to reflect the shoulder function for the week before the injury

#### Distribution of rotator cuff tear and occult fracture of the insertion site

The distribution of the target condition and final diagnoses of the included patients are presented in Table 4. A full-thickness rotator cuff tear was diagnosed in 38 (32%), and 46 (38%) had the target condition rotator cuff tear and/or occult fracture at its insertion. The patients with rotator cuff tear were older than those without a tear (median age 67 years and 51 years, respectively, *p* < 0.001), but there was no gender disparity (53% and 51% females, respectively). Two thirds of the patients with supraspinatus tears had increased fluid in the subacromial/subdeltoid bursa, whereas 9 (25%) had a supraspinatus defect also on the uninjured side. Two of the latter had neither bursa nor joint fluid on the injured side. Ten patients had full-thickness, partial-width subscapularis tears. Eight extended from a supraspinatus tear, only two were isolated tears of the upper rim.

Four patients had indeterminate ultrasound results for small full-thickness tears. One of these had an occult fracture of the greater tubercle on MRI and could be included in the test analysis (Table 5). The last three were not included in the test analyses for the relevant tendons (2 supraspinatus, 1 subscapularis) and were not classified as the target condition in Table 4.

**Table 4:** Target condition and alternative diagnoses in 120 patients primarily diagnosed as soft tissue injury with negative conventional radiographs

Target condition	Frequency (%)
**Supraspinatus tear**	36 (30)
Supraspinatus single tendon tear*	14 (12)
2-tendon tear: Supraspinatus + infraspinatus	12 (10)
2-tendon tear: Supraspinatus + subscapularis	3 (3)
3-tendon tear*: Supra- + infraspinatus + subscapularis	5 (4)
Supraspinatus tear + occult fracture of greater tubercle	1 (1)
Supraspinatus tear + occult fracture of lesser tubercle	1 (1)
**Infraspinatus single tendon tear**	0
**Subscapularis single tendon tear**	2 (2)
**Occult fracture at tendon insertion without tendon tear**	8 (7)
Occult fracture of greater tubercle	7 (6)
Occult fracture of proximal humerus	1 (1)
**Final diagnoses in patients without the target condition**	
Shoulder contusion and glenohumeral sprain/strain	48 (40)
Glenohumeral dislocation without RC tendon tear	13 (11)
Acromioclavicular joint injury	8 (7)
Occult fracture of scapula (1 neck, 1 acromion)	2 (2)
Occult fracture of lateral clavicle	1 (1)
Sternoclavicular joint injury	1 (1)
Long head of the biceps tear	1 (1)
**Total**	120

* Two in each group had suffered glenohumeral dislocation; *RC* rotator cuff

**Table 5:** Physical examination test effectivity in detecting full-thickness rotator cuff tears and/or occult avulsion fracture of the tendon insertion on the greater tuberosity

Index test	Target condition	TP	FP	FN	TN	Sensitivity % (95% CI)	Specificity % (95% CI)	PPV % (95% CI)	NPV % (95% CI)	DOR (95% CI)	Acc (%)	Test yield (%)
*Abduction*												
Inability to abduct > 90°	SSP/FRX	36	18	7	45	84 (69–93)	71 (59–82)	67 (57–75)	87 (76–93)	12.9 (4.8–34.2)	76	100 (90*)
Painful arc	SSP/FRX	3	18	4	26	43 (10–82)	59 (43–74)	14 (6–30)	87 (77–93)	1.1 (0.2–5.4)	57	53 (46*)
Strength	SSP/FRX	32	17	11	56	74 (59–86)	77 (65–86)	65 (55–75)	84 (75–90)	9.6 (4.0–23.0)	76	98
Resisted abduction pain	SSP/FRX	40	33	4	40	91 (78–97)	55 (43–66)	55 (48–61)	91 (79–96)	12.1 (3.9–37.4)	69	99
Hawkins	SSP/FRX	13	20	19	50	41 (24–59)	71 (59–82)	39 (27–53)	72 (66–78)	1.7 (0.7–4.1)	62	87
*External rotation*												
AROM reduced ≥ 20°	ISP/FRX	11	19	13	62	46 (26–67)	77 (66–85)	37 (24–51)	83 (76–88)	2.8 (1.1–7.2)	70	100 (88*)
Strength	SSP/FRX	31	15	13	58	70 (55–83)	79 (68–88)	67 (56–77)	82 (74–88)	9.2 (3.9–21.8)	76	99
	ISP/FRX	21	26	5	67	81 (61–93)	72 (62–81)	45 (36–54)	93 (86–97)	10.8 (3.7–31.7)	74	99
Small finger test	SSP/FRX	29	10	15	64	66 (51–80)	86 (77–93)	74 (61–84)	81 (74–87)	12.4 (5.0-30.8)	79	100
	ISP/FRX	20	20	6	74	77 (56–91)	79 (69–86)	50 (39–61)	93 (86–96)	12.3 (4.4–34.8)	78	100
External rotation lag sign	SSP/FRX	5	2	27	59	16 (5–33)	97 (89–100)	71 (34–92)	69 (65–72)	5.5 (1.0–30.0)	69	91 (79*)
	ISP/FRX	4	4	14	73	22 (6–48)	95 (87–99)	50 (22–78)	84 (80–87)	5.2 (1.2–23.4)	81	91 (79*)
*Test combinations §*												
Inability to abduct > 90° + small finger test	SSP/FRX	40	23	4	51	91 (78–97)	69 (57–79)	63 (55–71)	93 (83–97)	22.2 (7.1–69.3)	77	100
Inability to abduct > 90° + external rotation strength	SSP/FRX	41	25	3	49	93 (81–99)	66 (54–77)	62 (54–70)	94 (84–98)	26.8 (7.5–95.1)	76	100
Resisted abduction pain + small finger test	SSP/FRX	41	33	3	41	93 (81–99)	55 (43–67)	55 (49–62)	93 (82–98)	17.0 (4.8–59.8)	69	100
Resisted abduction pain + external rotation strength	SSP/FRX	42	37	2	37	95 (85–99)	50 (38–62)	53 (47–59)	95 (82–99)	21.0 (4.7–93.2)	67	100

*TP*, true positive; *FP*, false positive; *FN*, false negative; *TN*, true negative; *PPV*,positive predictive value; *NPV*, negative predictive value; *DOR*, diagnostic odds ratio; *Acc*, accuracy; *AROM*, active range of motion; *SSP*, supraspinatus tears, isolated and combined; *ISP*, infraspinatus tears, all combined with supraspinatus tears; *FRX*, occult avulsion fracture of the tendon insertion; *§* Pos if at least one of the two tests was positive; *Test yield calculation when including patients with recent glenohumeral dislocation in whom the test was stopped at shoulder level by the examining physician or not performed because of recent dislocation

### Test results

#### Abduction tests, painful Arc and Hawkins sign

**Fig. 2:**
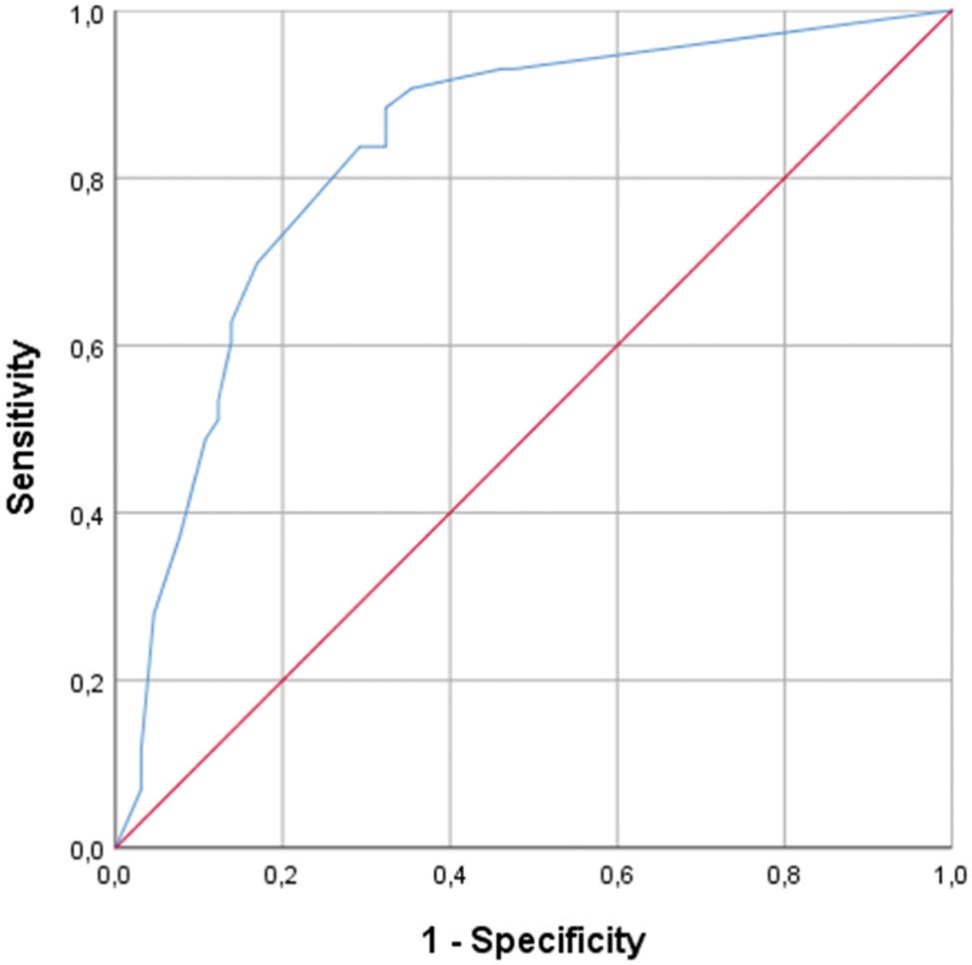
Receiver Operating Characteristic (ROC) curve for reduced abduction and target condition supraspinatus full-thickness tear with or without involvement of infraspinatus and/or avulsion fracture at insertion site. Area under curve (AUC) was 0.83

The inability to actively abduct the arm above 90° had the highest diagnostic odds ratio (DOR) for a single test of 12.9, whereas pain on resisted abduction had highest sensitivity of 91% (Table 5). The ROC curve analysis for loss of abduction is presented in Fig. 2. The results when not including avulsion fractures at the insertion site in the target condition were similar but slightly inferior (Appendix 4).

#### External rotation tests

The small finger test had the highest DORs of these tests due to high specificity (86%, Table 5), but the conventional strength test had higher sensitivity (70% vs. 66%) The specificity of the external rotation lag sign was ≥ 95%, but sensitivity was 22% or less.

#### Test combinations

The combination of the best tests related to abduction and external rotation (inability to abduct above 90° and the small finger test) was positive in 92% of the patients with a full-thickness supraspinatus tear, and 91% of those with such a tear and/or occult fracture of the greater tubercle (Table 5). The best cut off in a scoring system introducing age over 65 years as a third free variable was still at one of the two tests positive, irrespective of age (Appendix 5).

Only eight patients had all three tests as proposed by Murrell positive. Sensitivity was low (< 24%) and specificity high (> 95). In patients aged 60 years or more with two or three of the suggested tests positive as suggested by Murrell, the DOR was low (≤ 3).

## Discussion

The most important finding of the present study was that two simple tests; the inability to raise the arm above 90° and reduced strength in external rotation tested conventionally or with the small finger test, are valid clinical screening tests in the first line examination during the acute phase after shoulder injuries with negative x-rays. By discriminating between patients with and without a high probability of a tear of the upper rotator cuff, the tests may improve quality and save costs by selecting patients for imaging like ultrasound or MRI more accurately.

The results differ from previous studies on referred patients, where test efficacy is reported to be diverging and inconsistent [15, 16]. An association between the inability to abduct above shoulder level and rotator cuff tears has previously been demonstrated [8, 35], including in, to our knowledge, the only previous diagnostic study on acute rotator cuff tears [12]. By combining the inability to raise the arm above 90° and reduced external rotation strength, both high sensitivity and high specificity are taken into account, and two motions assessed in accordance with the rotator cuff muscles` combined function as dynamic stabilizers of the glenohumeral joint [48]. In a review of the clinical diagnosis of posterosuperior rotator cuff tears, the meta-analysis indicated that no single test could diagnose the condition sufficiently, and the authors recommend a combination of clinical tests, as also proposed in a previous level 1 diagnostic study of patients with full-thickness rotator cuff tears examined before surgery [49, 50]. In a previous study on the effect of lidocaine injections on shoulder tests after injury on the other hand, combining the tree most accurate tests; active abduction < 90°, Jobe supraspinatus test and either Hawkins test or the external rotation lag sign (ERLS), did not improve the diagnostic accuracy [12]. This may be due to a certain similarity between the Jobe test and active abduction < 90°, as well as the relatively low interrater reliability for the Hawkins test and ERLS in the present patient population [26].

It may be argued that diagnosing acute traumatic tears may push patients towards surgery, but the assumption of such tears being a key indication for surgery is controversial [31, 51–53]. A pivotal element for further studies on optimal treatment of acute rotator cuff tears, is to develop improved diagnostic pathways for the first line services where most patients are examined. Improved diagnostics would also benefit a shared decision approach to treatment.

### Acute tears versus asymptomatic chronic defects

The median age of the patients with rotator cuff tears was twelve years older than that of the total cohort. With increasing age there is also an increasing prevalence of asymptomatic rotator cuff tears [19, 22, 23, 54]. Bilateral imaging as in the present study has been suggested to deal with the problem of accidental findings of chronic tears [19]. Seven of the nine patients with a tear also on the uninjured side, had joint or bursa fluid in the injured shoulder, signs reported to be associated with acute injury [43]. The finding of an asymptomatic tear in the uninjured shoulder does however not preclude an acute tear or enlargement of a pre-existing tear in the affected shoulder. We have therefore adhered to the most used definition of an acute tear as described in the methods section [8, 30, 31].

### Other abduction tests

We registered abduction pain intensity at four levels. When transforming them into a binary test (positive = pain against gravity or isometric force), sensitivity and DOR were high. This is not entirely surprising considering the resemblance to the Supraspinatus/Jobe/Empty can test [14, 16, 55–57]. The test was performed in 30° abduction instead of 90° and did not involve internal rotation of the arm. This is less painful for the acutely injured patient and thereby makes the test more feasible for the examiner. Despite this, the ability to abduct above 90° or not may be easier to interpret.

### External rotation tests

Muscle strength evaluated by the small finger test was found to be as useful as the conventional muscle strength test and both were better than the external rotation lag sign. Interestingly, the first test was the most specific. In an earlier study the resisted external rotation test assessing muscle weakness was reported to achieve the highest area under the curve for detecting infraspinatus tendon tears [58]. In another study on test combinations to detect both full- and partial-thickness tears, external rotation strength was one of three signs along with age ≥ 65 and night pain, which in a scoring system best predicted a tear [59]. Interpreting external rotation also in relation to the supraspinatus, is supported by the combined function of the rotator cuff muscles [48].

The external rotation lag sign had high specificity but too low sensitivity to be recommendable in the present setting, similar to the findings in another recent study [56]. Also, we previously reported that reliability for the external rotation lag sign was only fair (Cohen`s Kappa 0.40) in the present patient population [26].

### Choice of tests and the DOR

In patients who have suffered acute shoulder trauma, tests should not depend on the patient being able to raise the arm, as many are unable to do so or get pain that interferes with test interpretation. Tests like the drop arm test [60], dropping sign [61], Patte`s test [62], full- and empty can [55, 60, 62] were therefore not included in the present study.

In the primary health care and emergency department setting, it may be argued that not missing the condition of interest (high sensitivity) is more important than high specificity. Rotator cuff tears and occult fractures are important to diagnose at an early stage in order to put the patient on the right treatment pathway from the start, but do not require immediate surgery. If tests are to be useful for screening high numbers of soft tissue shoulder injuries, specificity also needs to be considered to avoid high numbers of false positives requiring unnecessary ultrasound or MRI imaging. The DOR is therefore suitable for comparing test effectiveness in the present study, as it accounts for both the ability to recognize disease as well as the ability to exclude it.

### Test combinations

Thirteen tests were included, and exploring all possible test combinations was beyond the scope of the present study. We have chosen a pragmatic approach and provided data for some combinations of tests that were robust as single tests and could easily be performed and implemented in settings with acutely injured patients. We cannot exclude that other combinations may perform better statistically.

The test clusters for predicting full-thickness rotator cuff tears as proposed by Murrell for referred patients with shoulder pain [38], were not effective in the acute phase after shoulder trauma. Impingement is one of the three criteria, and neither the Hawkins nor the Painful arc tests [60, 63], were useful in predicting the target condition in the present setting.

### Strengths and weaknesses

The main limitation of the study is that results are only valid for patients over forty years that have follow-up within three weeks of an acute soft-tissue shoulder injury. More than half of the eligible patients were excluded, mainly due to ongoing neck-/shoulder pain at the time of injury. Whether the results are generalizable to this group needs to be investigated. The second, important limitation is that the confidence intervals were quite wide also for the best tests, indicating that a larger sample might have been preferable. Third, the number of subscapularis tears was low, consequently we were not able to evaluate tests for subscapularis tears. Tears of the upper rim may be difficult to detect by ultrasound and MRI [64–66], and the number may be underestimated.

The present study is to our knowledge the first presenting the accuracy and reliability of physical examination tests in non-referred patients after acute shoulder injury with negative X-rays. The study is aimed at emergency departments and primary health care, and the age and gender distribution indicate that we selected a representative cohort. The included tests reflect their usefulness in the emergency setting. Future studies may evaluate whether these tests are useful also in other hospital settings. The reliability of the tests has been published previously and the most accurate tests in the present study were also reliable [26]. As all patients underwent the ultrasound, the risk of verification bias present in studies with surgery as the gold standard, was avoided [16]. Different physicians blinded to the result of the ultrasound examination performed the tests. We screened the contralateral shoulder and adhered to the STARD statement [67, 68].

## Conclusions

In the present cohort of first line patients with acute soft tissue shoulder injury without ongoing neck and shoulder problems, two simple tests; the inability to abduct the arm above 90° and weakness in external rotation were effectively able to diagnose full-thickness tears of the upper rotator cuff and the integrity of its insertion. This may improve the quality of health care and provide a simple and cost effective tool for aiding the decision of which patients should be referred for MRI or ultrasound at the first follow-up.

## Electronic supplementary material

Below is the link to the electronic supplementary material.

Supplementary Material 1

Supplementary Material 2

Supplementary Material 3

Supplementary Material 4

Supplementary Material 5

## Data Availability

The data of the present study have been deposited according to the rules and regulations of Oslo University Hospital in the hospital`s research data repository, and is available from the corresponding author upon reasonable request.

## Footnotes

1 Invented by Peer H. Staff, former Head of Department of Physical Medicine and Rehabilitation, Oslo University Hospital.

## Abbreviations

AROM: Active range of motion
ERLS: External rotation lag sign
DOR: Diagnostic odds ratio
MRI: Magnetic resonance imaging
OSS: Oxford Shoulder Score
ROC: Reciever Operating Characteristic
SD: Standard deviation
STARD: Standards for Reporting Diagnostic Accuracy (2015)
