# Supplementary material for: Physical examination tests in the acute phase of shoulder injuries with negative radiographs: a diagnostic accuracy study
Source: BMC Musculoskelet Disord. 2025 Jun 3;26:546. doi: 10.1186/s12891-025-08754-1 (PMC12131457; doi:10.1186/s12891-025-08754-1)
Supplement: Supplementary file 1 — Supplementary Material 1 [file 12891_2025_8754_MOESM1_ESM.docx]

# FOLLOW-UP OF SOFT TISSUE INJURIES OF THE SHOULDER

(Excl dislocations)

### First visit

Patients with

- Negative conventional x-rays in two views

## and

- At least one of the following:
  - Active range of abduction reduced by > 30 degrees compared to the uninjured side by normal clinical examination, or significant loss of strength
  - Active range of external rotation reduced by > 20 degrees compared to the uninjured side by normal clinical examination, or significant loss of strength
  - Pain of 4 or more on a numeric rating scale

Should be offered follow-up 10 - 14 calendar days after the accident.
