# Supplementary material for: Physical examination tests in the acute phase of shoulder injuries with negative radiographs: a diagnostic accuracy study
Source: BMC Musculoskelet Disord. 2025 Jun 3;26:546. doi: 10.1186/s12891-025-08754-1 (PMC12131457; doi:10.1186/s12891-025-08754-1)
Supplement: Supplementary file 2 — Supplementary Material 2 [file 12891_2025_8754_MOESM2_ESM.docx]

| Appendix 2 Detailed description of clinical signs and tests for acute rotator cuff tear and/or avulsion fracture at insertion site | | | |
| --- | --- | --- | --- |
| Test | | **Description** | **Interpretation and registration** |
| Abduction | | | |
|  | AROM | Patient abducts both arms with thumbs up. Demonstrated in the scapula plane. (Stopped at shoulder level (90°) if glenohumeral dislocation). | Negative if > 90°, positive if ≤ 90°  Number of degrees |
|  | Painful arc | As above | Positive if painful arc between 60° and 120° with notably less pain in the rest of the arc, pain localized to deltoid region. Registered: pos, neg, not possible |
|  | Strength | 90° flexion in elbow, examiner alongside patient facing same way, abduction resisted by examiner, tested in both 0 and 30° | Normal if clinically indistinguishable to healthy shoulder  Normal or reduced |
|  | Resisted abduction pain | As above, arm in 30°- 40° abduction | Positive if i) or ii), negative if iii) or iv),  Registered as pain against: i) gravity alone, ii) isometric force, iii) eccentric force, iv) no pain |
|  | Hawkins | Shoulder and elbow in 90° flexion. Scapula stabilized. Internal rotation of upper arm by turning the elbow | Positive if pain provoked on internal rotation. Registered: pos, neg, not possible |
| External rotation | | | |
|  | AROM | Back against wall, elbows in 90° flexion, lower arm pointing forwards, thumbs up | Negative if < 20° degrees difference, positive if ≥ 20°  Number of degrees |
|  | Strength | Arm positioned as above, examiner facing patient applying internal rotation force on both distal lower arms | Normal if clinically indistinguishable to healthy shoulder  Normal or reduced |
|  | Small finger test | Arm as above, examiner on patient`s side, patient to resist internal rotation force by examiner`s 5^th^ finger to the distal forearm (ill. Fig 2) | Positive if patient cannot resist examiner`s force |
|  | External rotation lag sign | Examiner supports and elevates patient`s elbow to 20°, lower arm brought to maximum external rotation minus 5°, patient to hold position | Positive if unable to hold position. Registered: pos, neg, not possible |
| Internal rotation | | | |
|  | AROM | Hand on back | 0-plane, gluteal, lumbar or inter-scapular area |
|  | Belly-press | Patient presses flat hands against belly with elbow in forward position | Positive if elbow cannot be held in or if clearly reduced strength. |
|  | Internal rotation lag sign | Examiner extends and internally rotates arm with elbow in 90°, hand off back, patient to hold position | Positive if unable to hold position. Registered: pos, neg, not possible |
|  | Internal rotation lag sign mdf | Patient places flat hands against belly, examiner brings elbows in forward position | Positive if unable to hold position. Registered: pos, neg, not possible |

*AROM* active range of motion; *mdf* modified
