# Supplementary material for: Physical examination tests in the acute phase of shoulder injuries with negative radiographs: a diagnostic accuracy study
Source: BMC Musculoskelet Disord. 2025 Jun 3;26:546. doi: 10.1186/s12891-025-08754-1 (PMC12131457; doi:10.1186/s12891-025-08754-1)
Supplement: Supplementary file 3 — Supplementary Material 3 [file 12891_2025_8754_MOESM3_ESM.docx]

**Appendix 3**

**Fig.3** The age distribution in the study population compared to the general population of Oslo ≥ 40 years in 2016.
