# Supplementary material for: Physical examination tests in the acute phase of shoulder injuries with negative radiographs: a diagnostic accuracy study
Source: BMC Musculoskelet Disord. 2025 Jun 3;26:546. doi: 10.1186/s12891-025-08754-1 (PMC12131457; doi:10.1186/s12891-025-08754-1)
Supplement: Supplementary file 4 — Supplementary Material 4 [file 12891_2025_8754_MOESM4_ESM.docx]

| Appendix 4 Physical examination test effectivity in detecting full-thickness rotator cuff tears. Occult fracture at insertion excluded as target condition. | | | | | | | | | | | | |
| --- | --- | --- | --- | --- | --- | --- | --- | --- | --- | --- | --- | --- |
| **Index test** | | **Target condition** | **TP** | **FP** | **FN** | **TN** | **Sensitivity % (95% CI)** | **Specificity %**  **(95% CI)** | **PPV %**  **(95% CI)** | **NPV %**  **(95% CI)** | **DOR**  **(95% CI)** | **Acc**  **(%)** |
| Abduction | | | | | | | | | | | | |
|  | Inability to abduct > 90° | SSP | 29 | 24 | 6 | 46 | 83 (66-93) | 66 (53-77) | 55 (46-63) | 88 (78-94) | 9.3 (3.4-25.4) | 71 |
|  | Painful arc | SSP | 2 | 19 | 4 | 26 | 33 (4-78) | 58 (42-72) | 10 (3-26) | 87 (78-92) | 0.68 (0.1-4.1) | 55 |
|  | Strength | SSP | 25 | 23 | 10 | 57 | 71 (54-85) | 71 (60-81) | 52 (42-62) | 85 (77-91) | 6.2 (2.6-14.9) | 71 |
|  | Resisted abduction pain | SSP | 32 | 40 | 4 | 40 | 89 (74-97) | 50 (37-61) | 44 (38-51) | 91 (79-96) | 8.0 (2.6-24.7) | 62 |
|  | Hawkins | SSP | 8 | 24 | 16 | 53 | 33 (16-55) | 69 (57-79) | 25 (15-39) | 77 (71-82) | 1.1 (0.4-2.9) | 60 |
| External rotation | | | | | | | | | | | | |
|  | AROM reduced ≥20° | ISP | 7 | 22 | 8 | 67 | 47 (21-73) | 75 (65-84) | 24 (14-38) | 89 (84-93) | 2.7 (0.9-8.2) | 71 |
|  | Strength | ISP | 13 | 34 | 4 | 67 | 76 (50-93) | 66 (56-75) | 28 (21-36) | 94 (88-98) | 6.4 (1.9-21.1) | 68 |
|  | 5^th^ finger test | ISP | 13 | 27 | 4 | 75 | 76 (50-93) | 74 (64-82) | 33 (24-42) | 95 (89-98) | 9.0 (2.7-30.1) | 74 |
|  | External rotation lag sign | ISP | 3 | 5 | 8 | 79 | 27 (6-61) | 94 (87-98) | 38 (14-68) | 91 (87-93) | 5.9 (1.2-29.5) | 86 |

*TP* true positive; *FP* false positive; *FN*,false negative; *TN* true negative; *PPV* positive predictive value; *NPV* negative predictive value; *DOR* diagnostic odds ratio; *Acc* Accuracy; *AROM* active range of motion; *SSP* supraspinatus tears, isolated and combined; *ISP* infraspinatus tears, all combined with supraspinatus tears.
