# Supplementary material for: Physical examination tests in the acute phase of shoulder injuries with negative radiographs: a diagnostic accuracy study
Source: BMC Musculoskelet Disord. 2025 Jun 3;26:546. doi: 10.1186/s12891-025-08754-1 (PMC12131457; doi:10.1186/s12891-025-08754-1)
Supplement: Supplementary file 5 — Supplementary Material 5 [file 12891_2025_8754_MOESM5_ESM.docx]

# Appendix 5

To explore the influence of age on the main results, we performed logistic regression of a model with three free variables: the inability to abduct above shoulder level, the 5^th^ finger test and age. A ROC curve analysis was used to establish the optimal age cut-off point, which was 65 years. From the results of the logistic regression a scoring system from 0 to 5 points could be deducted for the probability of the target condition (Fig. 5). Each positive test scored two points, whereas age above 65 scored one point. Finally a diagnostic screening test evaluation of cut-off levels of the scoring system was performed in OpenEpi. The best cut-off point was between 2 and 3 points, meaning only one test needed to be positive irrespective of age.

|  | | B | Sig. | Exp(B) | 95% C.I.for EXP(B) | |
| --- | --- | --- | --- | --- | --- | --- |
|  |  |  |  |  | Lower | Upper |
| Step 1^a^ | Inability to abduct over 90° positive | 1.976 | .000 | 7.212 | 2.402 | 21.652 |
|  | 5th finger test positive | 1.974 | .000 | 7.198 | 2.490 | 20.806 |
|  | Age >65 | 1.283 | .025 | 3.607 | 1.172 | 11.098 |
|  | Constant | -2.720 | .000 | .066 |  |  |

**Table 6** Logistic regression of test combination, indicating one of the two tests positive should count as two points, and age > 65 as one point in a scoring system.
